# Publishing proteomic data

**DOI:** 10.1186/1477-5956-4-8

**Published:** 2006-04-28

**Authors:** Martin Latterich

**Affiliations:** 1 Department of Anatomy and Cell Biology, McGill University, Montreal, QC, H3A 2B2, Canada

## Abstract

Scientific publications should provide sufficient detail in terms of methodology and presented data to enable the community to reproduce the methodology to generate similar data and arrive at the same conclusion, if an identical sample is provided for analysis. The advent of high-throughput methods in biological experimentation impose some unique challenges both in data presentation in classical print format, as well as in describing methodology and data analysis in sufficient detail to conform to good publication practice. To facilitate this process, Proteome Science is adopting a set of methodology and data presentation guidelines to enable both peer reviewers, as well as the scientific community, to better evaluate high-throughput proteomic studies.

## Editorial

Historically, most scientific publications included a detailed methodology section that provided details on source of reagents, information, such as batch or lot numbers, and a description of methodology that would enable another research group to follow the same procedures. Given the same starting material, this practice would allow arriving at identical or very similar data. At the very least, methodology sections should refer to prior publications that provide sufficient experimental detail to allow the reproduction of scientific experiments. Most publications would then display "typical" results, such as photographs or micrographs of the experimental subject, images of detected molecules, or minimally processed data, such as statistically evaluated graphs or tables. These results were displayed together with negative and often positive controls that validate the experiment and reagents. The printed media was mostly adequate to publish these studies, because most studies investigated individual phenomena or molecules.

The advent of high-throughput methods in biological experimentation have imposed some unique challenges both in data presentation in classical print format, as well as in describing the methodology and data analysis workflow in sufficient detail to conform to good publication practice. This especially is an issue with proteomic analyses conducted by mass spectrometry [1,2]. Electronic media and public repositories are addressing the need for publishing uninterpreted data sets [3-5], such as raw or minimally processed mass spectrometer data, as well as lists of identified peptides. The remaining challenge is in the generation of ontologies and common experimental descriptions that capture the wealth of information that has both gone into the design and the analysis of proteomic experiments. This ultimately is needed when directly comparing multi-centre studies.

Much progress has been made by the community to propose data format standards that are compatible with most if not all analytical platforms [5,6]. However, there appears to be less conformity in the community when deciding what are minimal publication standards for such proteomic datasets [3]. The peer review process is normally rigorous enough to weed out submissions that are considered poor quality due to study design, choice of methods, or overall data quality. Unfortunately, dependent on the expertise of the peer reviewer, methodological detail is sometimes not considered as much as it should. While in the short term these studies will have their place in the community, in the long term they may not be considered valid because of lacking descriptive detail.

To ensure that studies of this nature withstand the test of time, Proteome Science has adopted a set of methodology and data presentation guidelines to enable both peer reviewers, as well as the scientific community, to better evaluate high-throughput proteomic studies leading to peptide and protein identification. These guidelines are by no-means top-down guidelines to restrict publication; instead, they are meant to reflect the accepted community standards in the field. As always with community guidelines, the publication guidelines proposed by the HUPO Proteomics Standards Initiative [7] will hopefully help to enable our authors to withstand the critique by the proteomics community over time. They are in no way intended to impose a standardized method to conduct experiments, which would be counterproductive to this still emerging and exciting field. We expect our authors to adhere to good scientific practise, such as listing source materials, methods of sample processing, the precise conditions to which samples were exposed prior to sampling, and the number of times an experiment has been conducted. In addition, if the authors use mass spectrometry to identify proteins in their samples, we recommend the adherence to the following guidelines to allow re-interpretation of the experimental data and comparison to other studies.

The following publication guidelines for the reporting and documentation of mass spectrometer-based peptide and protein identifications have in part been proposed by the HUPO Proteomics Standards Initiative [7,8]. They were heavily consulted during the development of publication recommendations for Proteome Science:

(a) Supporting information to be included in submitted manuscript:

1. Make, model, and version number of mass spectrometer, version of operating software, detailed acquisition parameters, and performance specs, such as resolution, sensitivity and dynamic range. If LC-MS/MS was performed, make, model, and version of HPLC system, operating specs on flow rate, gradients and columns used. Details on ionization source and conditions. Number of times experiment has been performed, and concordance between experiments.

2. The method(s), software (including version number) used to create the peak list from raw spectra, and the pertinent parameters used in the creation of the peak list. If custom algorithms or software were used to compile the list, these need to be listed in detail. Examples include parameters, such as smoothing, signal-to-noise ratio, whether charge states were calculated or peaks de-isotoped. In cases where additional customized processing of peak lists have been performed, such as clustering or filtering, the algorithm or software (including version) must be referenced or described.

3. The application and version number used for database searching, as well as the search parameters. Examples include precursor-ion mass tolerance, fragment-ion mass tolerance, fixed and variable modifications allowed for, number of missed cleavages, protein cleavage agents, isotopic or isobaric tagging chemistry, and so on.

4. The name and version of the sequence database and sequence space searched, including details on taxonomy and other search restrictions. If the database was custom compiled, a complete description of the sequence source is needed, and if not easily reproducible, a provision for making the database publicly available is needed. The number of entries actually searched from each database should be included. Authors should justify the use of very small databases, since this may generate misleading assignments. Common contaminants (keratins, trypsin) should be included in the database.

5. Methods used to interpret MS/MS data, thresholds and values specific to judging probability of identification, statistical methods used, and description of how analysis was validated, need to be described.

6. For large projects (e.g. mapping or comparison between complex fractions), additional statistical details should be listed, as they are pertinent to identification certainty, determination of false-positive rate, randomized database validation, or other computational approaches.

(b) When compiling information for protein identifications, the following information should be included:

1. Accession number and database source.

2. Score(s) and any statistical information for searches conducted.

3. Sequence coverage, expressed as the number of amino acids spanned by the assigned peptides by the intact protein's length.

4. The total number of peptides assigned to the protein. To compute this number, different forms of the same peptide are to be counted as one peptide.

(c) When presenting relative and absolute quantification data, the following information should be listed in addition to the information in section (b):

1. Quantification methods used and labelling conditions, if any.

2. Calibration standards used, if any.

3. Algorithms, software, and method details on how quantitative data was obtained.

4. Concordance and variance for different peptides from same protein, and method used to average individual peptides for a given protein.

5. Additional statistical information, such as p-values of quantified proteins, and common variance between experimental repeats.

## Competing interests

The author(s) declares that they have no competing interests.

## Authors' contributions

ML contributed 100 % to the Editorial Commentary.
